# Autonomous-submissive orientations and aggression of students at a metropolitan university in South Africa: Mental health implications

**DOI:** 10.4102/curationis.v43i1.2142

**Published:** 2020-11-03

**Authors:** Chris Myburgh, Marie Poggenpoel, Cornelius Fourie

**Affiliations:** 1 Department of Educational Psychology, Faculty of Education, University of Johannesburg, Johannesburg, South Africa; 2 Department of Nursing Science, Faculty of Health Sciences, University of Johannesburg, Johannesburg, South Africa

**Keywords:** aggression, autonomous-submissive orientation, descriptive, differences, exploratory, factor analysis, university students

## Abstract

**Background:**

Students are sometimes subjected to difficult circumstances to achieve success. Studying under these circumstances could cultivate aggression towards self and others, and the environment. Little, if any, published research is available dealing with students being orientated autonomous versus being submissive and perceptions of aggression.

**Objectives:**

To explore and describe the perceptions of groups of students being orientated autonomous versus students being submissive and perceptions of aggression. Recommendations concerning these two groups of students are made.

**Method:**

An exploratory quantitative research design that is descriptive and inferential in nature was applied. A questionnaire was electronically distributed to students in a faculty. The questionnaire consisted of items on biographic, personality and aggression. In the statistical analysis Cronbach’s alphas, principal component analysis were done and hypotheses were tested on differences between students orientated autonomous and those being submissive.

**Results:**

Of the 266 completed questionnaires used, 177 were received from females and 89 from males. Eighty-two (82) of these were honours, masters or doctoral students. Findings reflected graded differences between students being autonomous and students being submissive orientated concerning Overt verbal aggression (means below 1.82), Overt physical aggression (means below 2.53) and aggressive inclination towards others (means below 3.01). The implications are that students are to be sensitised to be reflective of their levels of aggression. University management should help students to be reflective concerning their aggression, and to establish congruency between self-perception and reality.

**Conclusion:**

Students should be helped to understand and manage their aggressive inclination.

## Introduction

Historically, tertiary institutions’ inclination towards students was elitist in nature (Stemen 2014). Ter-tiary training was reserved for a privileged minority of persons from society, with universities focussing on the cognitive development of a selected number of persons (students) with an almost meagre emphasis on affective challenges experienced by students and staff – worldwide, this narrow focus has changed over the last decade (Big & Tall 2010; ed. Rӧhrs 1987). Universities are no longer institutions catering solely for a selected small number of the population, as ‘widening participation’ has started to become the ‘norm’ for access to tertiary study (Vignoles & Murray 2016). Widening participation has emerged as a major policy concern in several national contexts. It is connected to longer histories over struggles for the right to higher education, to concerns for greater fairness in society and to ensuring that higher education is more equitable and inclusive (Connell-Smith & Hubble 2018). It is also shaped by the growing diversification of student constituencies that have resulted from higher education expansion over the later decades of the 20th century (Burke 2016). This new ‘norm’ means that universities are no longer elitist in approach but are popularised to serve populations at large. To illustrate this point, in the last couple of years the number of first-generation students has increased to become by far the majority group of the South African higher education student population (more than 70%) (USAF 2018). The industry also increasingly competes with universities in training persons for their purposes and specific needs – thus taking prospective students away from universities that are also at the same time competing with private universities. Furthermore, the international competition between universities in the global market increased through the implementation of various rating systems (such as Times Higher Education World University Rankings, QS, U21 & ARWU) makes it attractive to rather attend universities of ‘higher ranking’. To be aligned to these changes put enormous additional stress on staff and students and are superimposed on the existing challenges of coping with the new generation of students.

In South Africa, the changed foci of tertiary institutions are even more accentuated. With the onset of democracy in South Africa changes in universities’ managerial structures took place as part of the drive towards transforming society at large. It can be assumed that these changes have increased the coping demands on students and staff compared with what happened in the rest of the world. Many of the students who are first-generation students come from a previous disadvantaged section of the population (black African students comprise 79% of the first-generation students in 2018 – Universities South Africa [USAF] 2018). Presently most of the students are also challenged by poverty – students receiving National Student Financial Aid Scheme (NSFAS) funding from the South African Government constitute roughly 42% of all university, and 70% of all Technical and Vocational Education and Training (TVET) enrolled students – more than 650 000 students received NSFAS funding in 2019 (Carolissen 2020).

Furthermore, the certification process changed and is increasingly challenging to students and staff. In this con-text, a work situation at universities in South Africa is created in which lecturers are often exposed to perceived work overload and are trying to ‘survive’ in exercising their everyday work (Toerien 2015). The complexity and resultant stress embedded in this scenario are being increased through the fact that prospective graduates are aware of their rights and are challenging all study boundaries by enforcing demands, whether right or wrong. Most students are successful in their studies, whilst some are ‘ejected’ from the university system (Van den Berg 2018). Presently ‘ejecting’ a student happens through the identification of non-achievement, meaning that some students are unable to achieve success through completing certain curricular milestones within a preset and restricted time period. Assessed as being unsuccessful is enforced through implementing highly specific regulations and rules.

The complexity, diversity and changed features of universities in South Africa have paved a breeding ground for ‘organisation uncertainty’, that is, staff and students are not sure what next will be expected of them – this state of uncertainty could prime them to behave aggressively (Harati, Aslani & Ashkabasy 2018). This phenomenon was during the last decade illustrated through actions such as the ‘fees must fall’, and the ‘decolonisation’ demands from students. The ‘stressful’ scenario sketched above often leads to situations where aggression is prone to develop, not only at a macro-level but more specific within micro-levels of everyday interactions and communication – thus affecting the mental health of the involved persons.

### Aggression

It was already pointed out by Onukwufor (2013:62–64) a number of years ago that aggression’s influence is visible on almost every level of an individual’s life and in society – a fact that still holds (Kohn 1988). Aggression presents itself as a multifaceted phenomenon that is part of individuals’ everyday experiences. Aggression manifests in diverse formats, whether direct or indirect, intentional or unintentional, intense or less intense, verbal or non-verbal or physical, Overt or covert and many more manifestations. Usually another person is at the focus of intended aggression, but objects can also be the focus (Anderson & Huesmann 2003:298–299; Breet, Myburgh & Poggenpoel 2010:511–526; Myburgh, Poggenpoel & Tolsma-Hastings 2017). Sadock, Sadock and Ruiz (2017:2471–2504), as well as Bandura (1973:2) and Orton (1997:70), state that aggression is behaviour that includes actions intended to cause physical injury; verbal attacks, premeditated social exclusion of others; coercion; intimidation; and managerial styles that have harmful psychological consequences to those persons subjected to it (Sadock et al. 2017:2471–2504). Sadock et al. (2017) proceed by emphasising that these behaviours should not be underestimated, nor the effects thereof on the self-esteem, social status and the happiness of the involved persons. Breet et al. (2010:511–526) state that the prevalence and presentation of aggression are challenging to demarcate. Intellectually capable persons, such as university students, seemingly also promote and execute a more subtle and indirect expression of aggression compared with persons of a lesser intellectual capability (Anderson & Huesmann 2003:301).

The above explication motivates that aggression is mostly observed or acted behaviour. It is vital to take cognisance of the fact that it is an individual or a group of individuals, who is experiencing aggression or acting aggression. All human behaviour is motivated (Sadock et al. 2017:2471–2504), and therefore it is an indi-vidual who decides to act or who interprets that aggressive behaviour is manifesting. The implication hereof entails that decision-making by a human being is taking place when aggression is acted on or perceived in a situation or in communication.

### Decision-making in prospective aggressive situations and communication within a university context: Mental health implications

When a holistic approach is followed towards students in particular, or lecturers in general, specific implications come to the fore. A person perceives, interprets and acts upon a prospective aggressive situation, implying that the person’s body, mind and spirit are involved in acting upon an interpreted prospective aggressive situation. Any action that follows will influence the mental health of the person, whether it is a prospective road rage situation or merely interaction within the family context, or interaction with a colleague. It is, therefore, anticipated that the inclination of persons towards themselves and others could inform the direction and impetus of communications and actions.

The process of decision-making of an individual during a perceived or prospective aggressive situation could be complicated – the person who must make the decision/s is involved both within a specific situation and a specific context. Some of the considerations that play a role when a perceiving person ‘identifies and observes’ a prospective developing aggressive situation are as follows:

- an internal locus or an external locus of control used by the involved person (Piraino 2013);
- is it an implicit or explicit decision? is it Overt or covert? what value orientations are at play?
- is the responsibility for actions taken or ignored (Weiner 1995)?
- is the person passive or active in the aggressive situation?

Furthermore, are actions reflecting autonomous or submissive decision-making ignoring or considering other persons? enforcing own rights or conforming to others’ perceived expectations? and is the person(s) internally or externally orientated? The context plays an essential role in the perceptions of aggressive situations through peer influences such as the acceptance of peers’ rules, peer pressures, the importance of peers’ ideas and the willingness to comply with the demands of other persons. The importance attached to the self, as opposed to the importance that is attached to others, will influence the person’s perceptions concerning the interpretations of aggressive situations. Although all those mentioned above can and do play a role in decision-making during a prospective aggressive situation in a university context, the role of an autonomous versus a submissive approach is of central importance.

Universities worldwide are unanimous in their claims that they are assisting future leaders to develop their leadership potential. Leaders should be independent decision makers; however, during interactions in the classroom and on campus with lecturing staff there is subtly and even openly a demand that students are to submit to the rules of order in the process of interaction. This demand uncovers the interplay and force field of tension between autonomous and submissive behaviour requested and sometimes even demanded from students. This inherent tension between an autonomous and a submissive approach in prospective aggressive situations is the focus of this article. The researchers wondered if an autonomous versus a submissive orientation of students would play a role in their perceptions in aggressive situations and the possible effect thereof on students’ mental health. It is not possible to demarcate all considerations explicitly, as human interactions and actions are complex, non-linear, intricacies are mostly graded or shaded and are rarely at the extremes of possibilities. However, what is obvious is that a person’s orientation towards self and others are at play in developing aggressive situations.

In this study, on the one hand, autonomy is viewed as perceptions and behaviour that reflect dimensions such as being independent, self-directed, self-sufficient, self-governing, self-ruling, separate, sovereign and free. On the other hand, submissiveness entails perceptions and behaviour that are viewed as being obedient, passive, compliant, subservient, docile, even meek, dutiful and accommodative. However, despite intensive literature searches little, if any, recent and published research is available specifically dealing with the perceptions of aspects of aggression of students being orientated more autonomously versus students being orientated more submissively.

Overarching, how individuals perceive themselves will influence their perceptions of, and communication with, others in the situations and the environment in which they are. We anticipated that a more autonomous versus a submissive self-perception would influence perceptions, decisions and behaviour in situations where aggression manifests. We acknowledge that no person is only and solely either autonomous or submissive in assessing situations and that orientations can and do change. Persons’ perceptions and behaviour are blended between an autonomous and a submissive orientation. Although the orientation of a person in an aggressive situation given the above is central, we did not find any published research that shed light on this vital aspect of unpacking aggression in a university context.

## General hypothesis

The scenario above sets the scene of a stressful situation for students and lecturers. Lecturers experience stress from management, their peers and their students (Toerien 2015). Fortunately, lecturers are managing to a large degree the interaction with their students and assist them to still achieve under these stressful conditions. However, this involvement could often also provoke aggression. The mental health of both involved groups is often resultantly affected negatively. Aggression perceived and experienced by students and by lecturers in stressful situation is often inevitable. Although aggression is a common phenomenon, it is perceived and interpreted differently by individuals subjected to it in the same or similar situations. Students are usually coming from an intellectually gifted section of the population, and are thus more able to manage their perceptions and experiences of aggression. Although students are often autonomous in their behaviour, they seem to understand the demands of being submissive when required. The researchers wondered how students who are more autonomously orientated versus students who are more submissively orientated in their perceptions differ in their perceptions of various aggressive situations. The researchers could not find any published research on the possible differences between the perceptions of groups of students autonomously and submissively orientated in their perceptions of various aspects of aggression at a university and what can or should be performed to facilitate the mental health of the involved persons (students). In this context, our general hypothesis was that students more autonomously orientated versus students more submissively orientated in their perceptions will differ in their perceptions of various aspects of aggression.

## Objective

In this article, the researchers explore and describe the perceptions of groups of students being orientated more autonomously versus students being orientated more submissively and their perceptions of aspects of aggression. Recommendations concerning the facilitation of the mental health of these students are also made.

## Research design and method

An exploratory, deductive quantitative research strategy that is descriptive and inferential in nature was applied (Creswell 2018). A made-for-purpose questionnaire to explore students’ perceptions of aggression and other personal aspects was developed, taking the questionnaire of Buss and Perry (1992) as the point of departure. This questionnaire was electronically distributed to all students in a specific faculty at a metropolitan university during October–November 2018. The responses to the questionnaire items formed the data and were used in the further statistical analyses – the questionnaire is available on request. Items concerning the perceptions of students on aggression were identified from the questionnaire and subjected to principal component analyses (PCAs; factor analyses), reliability analyses and hypothesis testing. Hypothesis testing was performed through investigating the differences in aspects of aggression between the responses of the students perceiving themselves as being autonomous and the responses of the students perceiving themselves as being more submissive.

The ethical measures, design of the questionnaire, sample and population, validity and reliability, and the com-parative, exploratory inferential statistical analyses are described in the following sections.

### Population and sample

A total of 332 completed survey questionnaires were collected from all students coming from one small faculty (454 students were invited to participate in the study) at a metropolitan university in South Africa. The nature of the purposive sample entailed that no power calculation of sample size could be performed. The size of the sample was therefore determined by the number of students that responded on the invitation to participate to fill out the questionnaire.

### Questionnaire

The questionnaire items focus on three main domains, namely biographic questions, question items on perceptions concerning aggression and a variety of personality aspects relevant to perceptions of aggression. Biographic items addressed aspects such as gender, age, race, home language and year level of study.

This article researched the potential differences between the perceptions of aspects of aggression (as indi-cated by their responses to the different questions) of groups of students having a more autonomous orientation versus students having a more submissive orientation. Thus, items were purposefully selected that describe stu-dents’ perceptions of being autonomous-submissive orientated (independent variable) and aggression (dependent variables).

A total of 85 questions on these and other aspects concerning aggression formed the questionnaire items. In this article, the identified question items about students’ orientation and aggression are given in Tables 1–3. Each question item was assessed by the participants on a five-point Likert-type scale ranging from ‘extremely uncharacteristic of me’ indicated with a ‘1’ through ‘extremely characteristic of me’ marked with a ‘5’.

**TABLE 1:** Autonomous versus submissive orientation.

Question number	Item	Mean	SD
27.	I do what my peers tell me to do.	2.19	1.06
28.	I accept my peers’ rules (for example, if they tell me to be home at midnight to do my homework, I will do so).	2.45	1.23
86.	I (am prone to) give in to pressure from my peers.	2.07	1.06
87.	I surrender my ideas/will to others.	2.58	1.19
88.	I negate my own ideas in favour of other persons when I am pressured.	2.38	1.11
89.	I comply with what my peers require from me.	2.55	1.13
90.	I (am prone to) give in to requests from other persons.	2.82	1.10

Cronbach’s alpha: 0.771.

SD, standard deviation.

**TABLE 2:** Three factors describing aggression: Rotated component matrix.^a,b,c,d^

Question number	Item	Factors		
		1	2	3
Q29	I have trouble controlling my temper	-	0.686	-
Q37	I lose my temper for no good reason	-	0.698	-
Q45	Given enough provocation, I will hit another person	-	0.630	-
Q47	I get into arguments when people disagree with me	-	-	0.578
Q48	I yell at people for no good reason at all	0.507	-	-
Q52	I get the urge to trip other people	0.561	-	-
Q53	I tell false stories about people	0.738	-	-
Q55	I plan secretly to bother other people	0.626	-	-
Q56	I tend to shove (push) people when I am upset	0.588	-	-
Q57	I say bad things about people behind their backs	0.787	-	-
Q58	I call people negative names	0.726	-	-
Q62	I sometimes feel like a powder keg ready to explode	-	0.613	-
Q63	I sometimes push other people down to the ground	0.577	-	-
Q65	I try to influence people to dislike a specific person with whom I am angry	0.596	-	-
Q68	My friends say that I am somewhat argumentative	-	-	0.818
Q76	I sometimes become so mad that I tend to break things	-	0.650	-
Q77	I view myself as aggressive towards myself	-	0.675	-
Q82	Some of my friends think I am a hothead	-	-	0.687
Q85	I often find myself disagreeing with people	-	-	0.660

a Rotation method: Varimax with Kaiser Normalisation; explained variance 51.7%.

Component 2: 0.811 (6 items); and Component 3: 0.732 (4 items).

Rotation converged in 7 iterations.

b Kaiser-Meyer-Olkin Measure of Sampling Adequacy: 0.887.

c Bartlett’s test of sphericity: *p*-value = 0.000.

d Cronbach’s alpha: Questionnaire 0.897 (19 items); Component 1: 0.857 (9 items).

Component 2: 0.811 (6 items); and Component 3: 0.732 (4 items).

**TABLE 3:** Results of Student’s *t*-tests on the differences between the perceptions of a more autonomous and a more submissive group of students concerning aggression.

Question number	Item	Group	Mean	SD	*p*
**Factor 1: Overt verbal aggression**					
48.	I yell at people for no good reason at all	High	1.64	0.87	0.002**
		Low	1.35	0.64	
52.	I get the urge to trip other people	High	1.92	1.07	0.001**
		Low	1.52	0.87	
53.	I tell false stories about people	High	1.61	0.81	0.000**
		Low	1.24	0.62	
55.	I plan secretly to bother other people.	High	1.81	1.06	0.004**
		Low	1.45	0.93	
56.	I tend to shove (push) people when I am upset	High	1.78	1.11	0.001**
		Low	1.37	0.83	
57.	I say bad things about people behind their backs	High	1.68	0.89	0.003**
		Low	1.36	0.75	
58.	I call people negative names	High	1.82	0.92	0.000**
		Low	1.39	0.73	
63.	I sometimes push other people down to the ground	High	1.56	0.90	0.011*
		Low	1.30	0.76	
65.	I try to influence people to dislike a specific person with whom I am angry	High	1.69	0.99	0.003**
		Low	1.36	0.74	
**Factor 2: Overt physical aggression**					
29.	I have trouble controlling my temper	High	2.53	1.34	0.016*
		Low	2.15	1.28	
37.	I lose my temper for no good reason	High	1.97	1.02	0.014*
		Low	1.68	0.97	
45.	Given enough provocation, I will hit another person	High	2.26	1.31	0.016
		Low	2.05	1.22	
62.	I sometimes feel like a powder keg ready to explode	High	2.36	1.45	0.021*
		Low	1.98	1.33	
76.	I sometimes become so mad that I tend to break things	High	2.25	1.40	0.000**
		Low	1.60	1.13	
77.	I view myself as aggressive towards myself	High	2.64	1.37	0.003**
		Low	2.11	1.44	
**Factor 3: Aggressive inclination in relationship to others**					
47.	I get into arguments when people disagree with me	High	2.37	1.06	0.048*
68.	My friends say that I am somewhat argumentative	Low	2.12	1.19	0.222
		High	2.61	1.19	
82.	Some of my friends think I am a hothead	Low	2.74	1.35	0.033*
		High	2.49	1.20	
85.	I often find myself disagreeing with people	Low	2.19	1.22	0.105
		High	3.01	1.13	
		Low	2.81	1.30	

SD, standard deviation.

*N* = 227; higher *N* = 117; lower *N* = 110.

* , Significant at the 5% significance level.

** , Significant at the 1% significance level.

### Data collection

As is stated, a questionnaire was developed to explore students’ perceptions of aggression and other personal aspects. The initial questionnaire developed by Buss and Perry (1992) was taken as the point of departure for this process. This questionnaire was distributed electronically to all students in one faculty during October–November 2018. After these questionnaires were cleaned up, i.e. questionnaires must have been fully completed, 266 (of the 332 received back; i.e. a response rate of 80.1%) remained and the res-ponses to the selected items were used in the further analyses – this constituted the survey sample for this investigation. The demographics of the sample used in this investigation are that of the 266 completed questionnaires, 177 were from female students and 89 were from male students. Furthermore, 82 of the 266 participating students were busy studying towards an honours, masters or doctoral qualification. The remaining students were either undergraduates, or students doing their post-graduate certificate in education (PGCE) studies (184).

### Data analysis

Data were analysed by using the SPSS (version 25) software package and consisted of firstly assessing the relia-bility and validity of the received responses. Especially the Cronbach’s alpha and the results of the PCAs were used to assess validity and reliability. Theoretical and qualitative aspects of reliability and validity are discussed below. Once this assessment was performed, further analyses concerning descriptive statistics (mean and standard deviation) and various factor analyses were conducted. Hypothesis testing concerning differences between groups with respect to the various factors were carried out by using *t*-tests. *p*-Values of 0.01 (1%-level of significance) and 0.05 (5%-level of significance) were used to establish the significance of differences between groups. These are indicated Tables 1–3.

### Validity and reliability

Establishing validity and reliability (Taherdoost 2016) in this research was necessary because, although the concept of aggression is real, in our research experience it is somewhat ‘evasive’. First and foremost, extensive qualitative empirical research was conducted over more than two decades and through completing numerous research articles, dissertations and theses. From these research projects, and especially through previous qualitative projects, it was clear that aggression is a complex and challenging concept to uniquely define. In this research, participating persons were aware that aggression is commonly experienced by almost all persons in almost all situations where people interact and communicate. However, when one probes further, it will become clear that the demarcation and precise determination of aggression are somewhat evasive. Meta-synthesis and relevant literature controls followed initial research. During the initial research, several interviews having a qualitative focus were conducted. The outcomes of these interviews were presented at na-tional and international conferences and through publications in peer-reviewed journals. Despite all these con-tributions and literature searches, the concept of aggression remains blurred and fuzzy. The findings from the research, as mentioned above, and literature controls were built into the preliminary questionnaire.

### Ethical consideration

Ethical measures, such as autonomy, non-maleficence, beneficence and justice (City University of Hong Kong 2020; Dhai & McQuoid-Mason 2011), were adhered to throughout the research process. The participants were invited to participate in the survey voluntarily and to complete the questionnaire. They could withdraw from the investigation at any time. No data that could identify individual participants were included in the questionnaire. The Faculty Ethics Committee gave clearance for this investigation (ethics clearance number: 2013-018, updated in 2017), registered with the National Health Research Committee of South Africa (NHREC). The designated research official of the university also cleared the execution of this research project. All data from students were anonymously submitted electronically to the researchers and were analysed by the researchers on university se-curity-protected personal computers. Data were on password-protected computers in locked university offices and only accessed by the researchers or authors of this article. Benefits for the participants were that they had the opportunity to reflect on their behaviour, own experiences and the beha-viour of other persons (Universal Declaration of Human Rights 2006).

### Independent variable, dependent variables and differences

In the paragraphs that follow, the operationalisation of the independent variable (namely autonom-ous-submissive orientation) and the dependent variables (namely the three factors describing perceptions of aggression of students) are demarcated and described.

*Autonomous versus a submissive orientation (Table 1)*: Seven items were identified from the questionnaire that could describe the orientation of students. The data from this group of students concerning the calculated means and standard deviations on the items describing their autonomous-submissive orientations are presented in Table 1.

To demarcate the group of students perceiving themselves as submissive and autonomous, the fol-lowing process was followed: This was performed by adding the raw responses of students on the seven items indicated in Table 1 to get a total per individual. This action resulted in students having a self-perception count with a minimum of 7 (=7 items × 1, the minimum on each item) and a maximum of 35 (=7 items × 5, maximum). A histogram was then used to divide the group of 227 into two independent groups, namely a group of size 110 perceiving themselves as being more submissive (having a total count between 7 and 17) and a group of size 117 perceiving themselves as being more autonomous (having a total count between 18 and 35).

The division was made to identify those persons who will normally act autonomous or submissive; ignore or consider other persons, or demand to enforce their rights or conform to others’ perceived expectations. Our previous research showed that persons who act upon perceived aggressive situations usually differentiate between the boundaries of either being autonomous or being submissive. It is anticipated that within the differ-ent contexts of a student’s situation, this would undoubtedly entail an orientation towards aspects such as the role that peers may play in personal decision-making, the acceptance of peers’ rules, the impor-tance of peer pressures, the importance of peers’ ideas and the degree of willingness to comply with another person’s demands, or to stand one’s own. The importance attached to the self versus the importance attached to others may also play a role in one’s perceptions concerning prospective ag-gressive situations.

The perception of the levels of aggression of these two groups is compared in the following section.

*Factors describing aggression (Table 2)*: To assess validity, the items that could indicate percep-tions of aggression were identified in the questionnaire. These items were then subjected to a PCA (factor analy-sis). Items with a factor loading of < 0.5 were removed from all further analyses. Furthermore, the relia-bility of each factor was assessed by using the calculated Cronbach’s alpha. Ill-aligned items were then removed from further analyses. The Kaiser-Meyer-Olkin (KMO) Measure of Sampling Adequacy of the factor anal-ysis on aggression (see Table 2) is 0.887 and is assessed as ‘great’ (see Field 2017). Also, Bar-tlett’s coefficient to assess sphericity was significant, with a *p*-value of 0.000 (Field 2017). Thus, conducting this PCA was considered as appropriate for this investigation. Finally, the ex-plained variance of the factor analysis is 51.7%. The conclusion to use these factors as a basis for the further assessment of differences between groups being more submissively and being more autonomously orientated (see Table 1) regarding their perceptions of aggression is therefore in line with the exploratory and tentative nature of this investigation.

Table 2 presents the rotated component solution of the factor loadings on the three identified factors and Cronbach’s alpha coefficients, KMO values and Barlett’s test *p*-values. Factor 1 was identified as *Overt verbal aggression*. This concept is based on indications that the items loading on this factor refer to planning and actions to act aggressively in primarily an Overt verbal manner, such as visible, unconcealed, open, plain and blatant verbal behaviour towards other per-sons. Factor 2 was identified as *Overt physical aggression* as items loading on this factor indicated aggressive behaviour that reveals open, blatant, evident, visible, unconcealed, explicit aggressive behaviour. There is an aggressive physical focus in the items loading on this factor. Factor 3 was identified as *Aggressive inclination concerning others* as items loading on this factor reflects aggressive beha-viour that is directed or concerned with interrelationships.

## Findings of the study

As this research is exploratory in nature, it was decided to focus on the description of the general trends in the data, rather than on minute details as shown in Table 3. The identified general trends also form the basis of the discussion of the implications of this research.

From the results of the factor analysis, the three identified factors on aggression were used in the further ana-lyses. The three factors described the different domains of aggression and formed the dependent variables for the investigation of differences between the more submissive and the more autonomous group of students. Because of the exploratory nature of this investigation, and the lack of any published research concerning the perceptions of own orientation of students and their perception of aggression, the researchers decided to follow a research approach that will only identify gross trends rather than to explore minute details in these data. Thus, only substantial and statistically significant differences between the responses of the compared groups are described. In Table 3, the means, standard deviations and *p*-values of the comparisons per item between the autonomous (high values) and the submissive (low values) groups as per one-sided Student’s *t*-test are given. The items are organised according to the three factors and will be discussed accordingly.

*Overt verbal aggression (Factor 1)*: It follows from the content shown in Table 3 that in the case of Overt verbal aggression, the mean per item of autonomously orientated (high) students is significantly higher than those of submissively orientated students (low) on ALL items loading on this factor. Furthermore, what is observed is that the means of both groups of students on a five-point scale indicate that students are in general not extremely aggressive and are seemingly somewhat submissive (all below means are 1.92).

It follows from these observations that the autonomous group of students are Overt verbally significantly more aggressive than the submissive group of students.

*Overt physical aggression (Factor 2)*: It follows from the content shown in Table 3 that as for Overt physical aggression, the mean per item of autonomously orientated students (high) is significantly higher than those of submissively orientated students (low) on most of the items loading on this factor (exception is item 45). Most of the differences are less prominent compared with factor 1 (in the case of factor 2, most of the *P*-values are on the 5%-level of significance). It is further observed that the means of the items loading on this factor for both groups of students (measured on a five-point scale) are small, indicating that the students are not extremely aggressive and are seemingly somewhat submissive (all means are below 2.64).

It follows from these observations that the autonomous group of students are Overt physically significantly more aggressive than the submissive group of students, but these findings are less prominent compared with the findings of factor 1 (Overt verbal aggression); that this difference in spite of the significant finding is even smaller than in the case of factor 1.

*Aggressive inclination about others (Factor 3)*: It follows from the content shown in Table 3 in terms of aggressive inclination concerning others (factor 3), the differences between the item means for autono-mously orientated students (high) and submissively orientated students (low) are less prominent. Differences, where they occur, are on the 5% level of significance – and for only two of the four items. Means are also relatively small (all above 2.12, but still below 3.01 on a five-point scale). Again, this group of students perceive themselves as relatively non-aggressive.

It follows from the above observations that the autonomous group of students seems to be Overt physically more aggressive than the submissive group of students; that the findings of factor 3 are less prominent than in the case of factor 1 (Overt verbal aggression); that this difference observed in factor 3 is, despite the significant finding, even smaller than those observed in the case of factor 1; and that this group of students seemingly perceive themselves as relatively non-aggressive concerning Overt physical aggression, but somewhat more Overt physical aggressive in the case of verbal aggression (factor 1 – slightly larger means).

*Overall findings indicate*: The hypothesis that there is a difference between the perceptions of students that are submissively orientated and autonomously orientated is supported, but this is a blended support as there are differentiations:

- Concerning Overt verbal aggression, this hypothesis is supported; all means are lower than 1.92 on a five-point scale.
- Concerning Overt physical aggression, this hypothesis is also supported (although less strongly as in the case of Overt verbal aggression); all means are < 2.64 on a five-point scale.
- Concerning aggressive inclination concerning others, this hypothesis is not strongly supported; all means are lower than 3.01 on a five-point scale.
- It seems that the students perceive themselves as low on aggression (low means), but that they perceive themselves as having a higher aggressive inclination concerning others than being Overt physically and Overt verbally aggressive.

## Implications and recommendations

The fact that there are definite differences between the perceptions of students inclined more autonomous and the responses of students with a more submissive orientation concerning various aspects of aggression indicates that students perceive various aspects of aggression implicitly different. As aggression in South Africa is widespread, this university and more specifically this faculty must interrogate the implications of this preliminary, but an important finding. Students should be assisted to identify, understand and manage their aggression. The observation that two of the three factors have an Overt focus on aggression, whether verbal or physical, might be indicative of what is happening in the rest of South Africa. Students should be assisted to evade acting Overtly aggressive and should instead attempt to channel their aggressive energy in constructive ways, such as reflection; debating issues actively; respecting themselves, their fellow students, their fellow South Africans and their fellow Africans; and through participating in sport.

Students’ self-perception that their levels of aggression are low is not always a true reflection of what is observed in society. This finding might be indicative that students’ perceptions of aggression are not a precise reflection of experienced and observed ‘real’ aggression. Students’ mental health can, therefore, be at risk as they are not able to identify and precisely demarcate their own inner life. Therefore, university management is obliged to assist students to understand, describe and manage their le-vels of aggression. This act is especially crucial, seeing that Foucault (1982) already stressed in 1982 that aggression is inherently power relationships that are at play; in other words, it is not a new phenomenon that must be discovered and addressed. This aspect and the influence thereof on the mental health of the students have not been interrogated in-depth in this study and should be further explicated in follow-up research.

Although somewhat disguised, an aggressive inclination towards other persons is present. Radwan (2015) and the Valley Behavioral Health System (2015) hold the view that this issue cannot be ignored. The findings from this study indicate that internal processes are at play in the levels of aggression that students show. If this observation is linked with the notion that all behaviour is motivated (Cherry 2015; Theories on Motivation 2015), a possible programme addressing the mental health of students to reflect and explicitly identify, demarcate and address their own aggression can be of value. Reflection by individuals on their thoughts and behaviour concerning their aggression might also facilitate their mental health. Add to this the reality that universities are places where stress often reaches prominent levels, and it becomes clear that universities can become places of turmoil if stu-dents’ levels of aggression are not addressed and restricted.

In university situations, in general, cognitive distancing is often at the order of the day and affective development is often underplayed. Students are not only intellectual beings – they are also spiritual beings. A holistic approach that encompasses and acknowledges that the body, mind and spirit of students are important is imperative (Magna Report 2016). Emotions, affection and a definite inclination towards self can be used to counteract aggression towards oneself (Psychlopedia 2015).

To facilitate the mental health development of these students, a psycho-educational programme can be developed. However, it should not be a specific programme, but rather an inherent programme that facilitates the integration of body, mind and spirit in an overarching approach aimed at facilitating the mental health of individuals and groups in an often clinical and cold university atmosphere (Magna Report 2016). In the case of the research group for this study, it should, therefore, form part of the ‘hidden curriculum’ in this faculty and university. Departments, faculties and the university should aim to become caring facilities. When lecturing staff and administrative staff become more caring towards the students they serve, experienced aggression in universities might be changed, and universities might even become places where aggression is managed healthily.

This scenario demands that this university should rethink its approaches towards students, and in doing so, that students’ needs can no longer being ignored or challenged. Holistic support systems to address the needs of challenged students and staff are imperative as the mental health implications thereof are clear on scrutiny of the fundamental domains it entails (Magna Report 2016). Therefore, further research using random samples from the whole spectrum of universities and faculties is essential.

## Limitations

Although some interesting findings came to the fore in the research leading to this article, they must be gauged with the utmost caution. This investigation was conducted at one metropolitan university in one faculty. Thus, no generalisation to the whole university system in South Africa can, or should, be inferred. Furthermore, males who participated in this investigation were in the minority. Although electronic investigations are becoming more prominent, there are definite shortcomings to the findings from such investigations. The use of self-perception instruments in assessing aggression have also demonstrated shortcomings, as no person, despite evidence to the contrary, would like to describe themselves as extremely ‘aggressive in orientation’.

## Conclusion

Aggression is part and parcel of this university’s set-up, although in our view maybe somewhat un-derplayed in the self-perceptions of the participating students. To simply ignore the (hidden) role of student ag-gression in this institution can be too disastrous to contemplate. Underlying all the findings as mentioned above is that, if specific cues are mindlessly ignored, aggression is fuelled. There is therefore an urge to act mindfully when the aggression thermostat is indicating that levels of aggression are increasing.
